# Edema Induced by a *Crotalus durissus terrificus* Venom Serine Protease (Cdtsp 2) Involves the PAR Pathway and PKC and PLC Activation

**DOI:** 10.3390/ijms19082405

**Published:** 2018-08-15

**Authors:** Caroline R. C. Costa, Mariana Novo Belchor, Caroline F. B. Rodrigues, Daniela de Oliveira Toyama, Marcos A. de Oliveira, Danielle P. Novaes, Marcos Hikari Toyama

**Affiliations:** 1 Institute of Biosciences, Coastal Campus, BIOMOLPEP, São Paulo State University (UNESP), 11330-900 São Paulo, Brazil; carolsbert@gmail.com (C.R.C.C.); belchor.mariana@gmail.com (M.N.B.); gaveira@yahoo.com.br (D.d.O.T.); danipnovaes2012@gmail.com (D.P.N.); 2 Instituto Butantan, 05503-900 São Paulo, Brazil; cfabri3@gmail.com; 3 Institute of Biosciences, Coastal Campus, LABIMES, São Paulo State University (UNESP), 11330-900 São Paulo, Brazil; scaffix@gmail.com

**Keywords:** snake venom serine protease, *Crotalus durissus terrificus* (Cdt), edema, inflammation, oxidative stress, protease-activated receptor, COX-2, MDA

## Abstract

Snake venom serine proteases (SVSPs) represent an essential group of enzymatic toxins involved in several pathophysiological effects on blood homeostasis. Some findings suggest the involvement of this class of enzymatic toxins in inflammation. In this paper, we purified and isolated a new gyroxin isoform from the *Crotalus durissus terrificus* (Cdt) venom, designated as Cdtsp 2, which showed significant proinflammatory effects in a murine model. In addition, we performed several studies to elucidate the main pathway underlying the edematogenic effect induced by Cdtsp 2. Enzymatic assays and structural analysis (primary structure analysis and three-dimensional modeling) were closely performed with pharmacological assays. The determination of edematogenic activity was performed using Cdtsp 2 isolated from snake venom, and was applied to mice treated with protein kinase C (PKC) inhibitor, phospholipase C (PLC) inhibitor, dexamethasone (Dexa), antagonists for protease-activated receptors (PARs), or saline (negative control). Additionally, we measured the levels of cyclooxygenase 2 (COX-2), malondialdehyde (MDA), and prostaglandin E2 (PGE2). Cdtsp 2 is characterized by an approximate molecular mass of 27 kDa, an isoelectric point (pI) of 4.5, and significant fibrinolytic activity, as well as the ability to hydrolyze Nα-benzoyl-l-arginine 4-nitroanilide (BAPNA). Its primary and three-dimensional structures revealed Cdtsp 2 as a typical snake venom serine protease that induces significant edema via the metabolism of arachidonic acid (AA), involving PARs, PKC, PLC, and COX-2 receptors, as well as inducing a significant increase in MDA levels. Our results showed that Cdtsp 2 is a serine protease with significant enzymatic activity, and it may be involved in the degradation of PAR1 and PAR2, which activate PLC and PKC to mobilize AA, while increasing oxidative stress. In this article, we provide a new perspective for the role of SVSPs beyond their effects on blood homeostasis.

## 1. Introduction

Some studies showed that the venom of *Crotalus durissus* ssp., including *Crotalus durissus terrificus* (Cdt), contains two serine-protease fractions, in addition to gyroxin. Both fractions are catalytically active, and are capable of degrading fibrinogen. *Crotalus durissus collilineatus* venom revealed the presence of two serine-protease fractions that were able to induce mild inflammation (edema), and the results suggested that their activity did not involve the presence of gyroxin. Based on their activity on synthetic chromogenic substrates, both fractions were classified as thrombin-like enzymes without any detectable plasmin-like activity, having a significant activity on S-2238 (a substrate specific for thrombin) and residual activity on S-2251 (a substrate specific for plasmin and streptokinase) [1]. 

Molecular modeling and structural comparisons of this simile gyroxin protein with other highly conserved serine proteases revealed that this gyroxin simile isolated from *Crotalus durissus collilineatus* comprised several common domains found in various thrombin-like enzymes of other poisons [1,2]. In addition, serine proteases isolated from *Crotalus durissus cumanensis* venom also showed the presence of two serine-protease fractions through a combination of molecular exclusion on a Sephacryl S-200 column and reverse-phase HPLC on a semi-preparative C8 column [3]. In both cases, this new fraction was found to be structurally different from gyroxin purified from the venom of Cdt. In addition, both fractions were eluted together with the crotoxin. These two enzymes are single-chain proteins of similar molecular masses that induce the coagulation of plasma. In terms of their biochemical characteristics, these enzymes are similar to other snake venom thrombin-like serine proteases and fibrinogenases, with characteristics unlike gyroxin. In both cases, these serine proteases were isolated in the fraction corresponding to gyroxin, while other enzymes were isolated in crotoxin (Figure S1). Furthermore, crotoxin, in addition to being the major fraction of *Crotalus durissus* ssp., revealed the presence of crotoxin B (CB), and also contained a crotapotin fraction with essential serine proteases.

Snake venom serine proteases (SVSPs) play a crucial role in the pathophysiological activity induced by venom. These classes of toxins represent an essential tool for the capture of prey using envenomation, on the basis of pivotal toxins being responsible for several blood homeostasis disorders during a snake venom bite, leading to major toxicity for some species. Since SVSPs are proteins that modulate blood homeostasis, several studies revealed their potential therapeutic use in the treatment of blood coagulopathy [2,4,5]. All SVSPs identified to date present a catalytic triad comprising histidine (His), aspartate (Asp), and serine (Ser), essential for their enzyme activity [2,5]. Recent studies strongly suggested that serine proteases BpirSP27 and BpirSP41, purified from *Bothrops pirajai* (Bpir), were able to activate the complement system, potentially inducing edema under experimental conditions, and may include proinflammatory features, as well as being significantly inhibited by nonsteroidal anti-inflammatory drugs [6,7,8]. Other SVSPs purified from *Bothrops alternatus*, *Bothrops mooeni* [9], *Trimeresurus elegans*, and *Trimeresurus jerdonni* venom revealed the ability to release kinin, as well as fibrinolytic and edematogenic activities [10].

Collectively, these studies suggest that these proteins should be included in a new class of proinflammatory toxins, akin to snake venom metalloproteases and secretory phospholipase A2 [11]. The results of the work presented herein showed that the venom of some *Crotalus durissus* ssp. contain serine proteases other than gyroxin, with samples showing strong fibrinolytic activity and amidolytic activity. Furthermore, some of the isolated enzymes were present in the crotoxin fraction, with the isolated SVSPs also capable of inducing edema. However, the proinflammatory effects and the possible mechanisms or pathways involved in inflammation induced by SVSPs remain unclear and need to be investigated [3,11]. Studies involving Cdt secretory phospholipase A2 (Cdt sPLA2) showed that arachidonic acid (AA) metabolism plays an essential role in edema and causes eicosanoid production [12,13]. Eicosanoids derived from AA exert a complex control of edema progression, since their production is considerably increased during inflammation, and their products are involved in pathogenesis related to the inflammatory process [12,13].

Cyclooxygenases 1 and 2 (COX-1 and COX-2) stimulate prostaglandin (PG), prostacyclin (PGI), and thromboxane (TX) production [14,15,16]. However, calcium-dependent cytosolic phospholipase A2 (cPLA2) is regulated by changes in intracellular calcium (Ca^2+^) levels through phosphorylation in response to various cellular stimuli [17]. The cPLA2 enzyme contains several crucial phosphorylation sites including Ser505, Ser727, and Ser515, which are phosphorylated by mitogen-activated protein kinases (MAPKs). Several PLA2 myotoxic PhTx-II (*Porthidium hyoprora* PLA2 II toxin) residues are also conserved across other PLA2 enzymes, namely Leu2, Phe 3, Ile 9, Thr 22, Cys 29, Cys 45, and Ala 93, whose hydrophobic side chains form the walls of a hydrophobic channel involved in substrate recognition and binding [17]. Other cPLA2 enzymes include diacylglycerol (DAG), which is involved in protein kinase C (PKC) and phospholipase C (PLC) activation. This activation is responsible for the production of inositol triphosphate (IP_3_) and DAG [18,19,20,21]. Notwithstanding, PKC and PLC are also key elements in pathway signaling mediated by protease-activated receptors (PARs). PARs are a unique family of G-protein-coupled receptors that play critical roles in homeostasis, thrombosis, and inflammation. These receptors are activated by numerous proteases [22], resulting in the mobilization of the G-protein and the activation of PKC and PLC [23]. Moreover, stimulation of cPLA2 generates two important compounds, lysophospholipids and AA, which are substrates for cyclooxygenases and lipoxygenases. The most stable product derived from the arachidonic pathway is prostaglandin E2 (PGE2). In addition, the presence of cell-surface receptors represents another essential feature of cPLA2,which stimulates the signaling pathways associated with the activation of protein kinases and the production of reactive oxygen species (ROS) [6,16,24]. 

During inflammation, AA metabolism itself may induce oxidative stress and lipid peroxidation, which increases the generation of products such as malondialdehyde (MDA), as well as new epitope capable of potentially inducing undesirable biological responses. These data suggest that the role of serine proteases is not restricted to their thymine function. Instead, they can also be essential modulator agents for blood homeostasis (e.g., SVSPs). These enzymatic toxins could also play a crucial role in AA mobilization, and they may induce inflammatory processes, which generate prostaglandins (PGs) [12,25,26]. The aims of this study were to structurally analyze a new class of SVSPs found in Cdt venom (Cdtsp 2), which reveals a trypsin-like activity, and to characterize the edematogenic effect of the novel serine protease isolated from Cdt venom. Additionally, we proposed an initial outline of the mechanism and signaling pathways involved in the edematogenic activity of Cdtsp 2, as well as the inclusion of SVSPs in a new class of edematogenic toxins alongside sPLA2 and venom metalloproteases (MPs).

## 2. Results

### 2.1. Purification of Cdtsp 2 and Coagulation Activity

**Figure 1:**
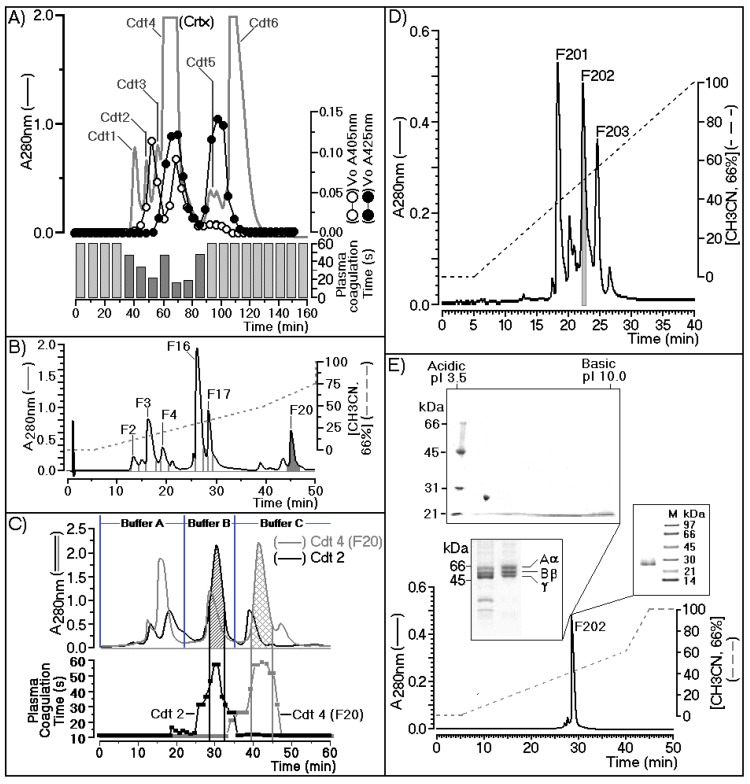
(**A**) Purification of fraction 202 (F202) through three steps of size exclusion chromatography (**A**) followed by Reverse phase HPLC in 5C columns (**B**), ion exchange (**C**), and reverse-phase HPLC in C4 columns (**D**). Its main biochemical characterization was done using two-dimensional electrophoresis (**E**).

Figure 1A shows the chromatographic profile describing the fractionation of *Crotalus durissus terrificus* venom using a Superdex G75 (1.6 × 60 cm) column previously equilibrated with ammonium bicarbonate buffer (0.1 M, pH 7.9), with isocratic flow maintained at 0.2 mL/min. The fractions were collected at 2-min intervals. The chromatographic run was monitored at an absorbance of 280 nm (A280) and each fraction collected were assayed for secretory phospholipase A2 using chromogenic substrate 4-nitro-3-benzoic acid (NOB) monitored at 425 nm (A425). The serine protease assay was determined using Nα-Benzoyl-l-arginine 4-nitroanilide hydrochloride as the chromogenic substrate (BAPNA) monitored at 405 nm (A405) and plasma coagulation were also monitored and presented in Figure 1A. In this figure, we showed that crotoxin fraction (Crtx; Cdt4) showed the presence of both enzymatic activity (PLA2 and serine protease) and significant plasma coagulation time. Cdt4 (Crtx) was applied on reverse phase HPLC column using a C5 wide pore (Sigma Aldrich) eluted with a discontinuous gradient concentration buffer B (Acetonitrile, CH3CN 66%, in TFA 0.5%). 

Under this condition, Cdt4 was fractioned in crotapotin (F2, F3, and F4), besides, secretory phospholipase A2 (F16 and F17) and F20 showed trypsin like enzymatic activity (Figure 1B). The F20 fraction was inhibited by trypsin, *N*-tosyl-l-phenylalanine chloromethyl ketone (TPCK), and Nα-tosyl-l-lysinyl-chloromethylketone (TLCK). However, sPLA2 (F16) was inhibited by its natural F3 Crtp inhibitor, the palmitoyl trifluoromethyl ketone inhibitor (PACOCF3, herein called PCF3), and the 5-(4-benzyloxyphenyl)-4S-(7-phenylheptanoylamino) pentanoic acid inhibitor (KH064), which had no effect on the F20 fraction as shown in Supplementary Figure S2. These data provide more information about positive identification of sPLA2 determined by NOB and crotapotin by its ability to decrease enzymatic of both fractions F16 and F17 and negative inhibitory of F20 fraction. All fractions were collected, pooled stored until lyophilization for further molecular steps.

F20 fraction purified from Cdt4 (Crotoxin) after freeze drying, showed a significant molecular stability and enzymatic activity. Cdt4 fraction, which corresponds to the second serine-protease fraction, was applied to a DEAE-5PW (Supelco) ion exchange column. The fractionation was performed on a column previously equilibrated with 0.05 M ammonium bicarbonate buffer (pH 7.9), and it was completed using three steps (buffer A, 0.05 M ammonium bicarbonate; buffer B, 0.2 M ammonium bicarbonate; and buffer C, 0.5 M ammonium bicarbonate). A final elution was made with 1 M ammonium bicarbonate, which showed no fractions. Under this condition both fractions provide from the size exclusion chromatography were subjected to DEAE-5PW chromatography following the elution protocol described elsewhere and the gray line showed the fractionation of F20 (Cdt4 F20) as well as Cdt 2 was appears as black line in the Figure 1C. This panel indicates the main serine protease active fraction that in case of fractionation of Cdt4, F20 appears as a gray grid, whereas Cdt2 appear as black grid peak (Figure 1C). Both fractions were found in Cdt4, F20 as well as Cdt2. Therefore, the biological effects induced by Cdtsp 2 did not involves contamination with sPLA2. Despite this, only the Cdt4 F20 fraction exhibited coagulation of citrated ox plasma performed on the basis of the study described by Fonseca et al. [27]. As shown in Figure 1C, both Cdt2 and Cdt4 exhibited one major serine-protease fraction. 

In the case of the fractionation of Cdt2, the serine protease was eluted with buffer B, and corresponded to gyroxin. The Cdt4 F20 fractionation also showed one major serine-protease fraction, which was eluted with buffer C. Therefore, under the same chromatographic conditions, we clearly showed that *Crotalus durissus terrificus* snake venom has two distinct serine-protease fractions. After the second step, the main serine-protease fraction purified from Cdt4 during the ion exchange was pooled, lyophilized, and subjected to a final chromatographic step performed using C4 reverse-phase HPLC. 

In Figure 1D, we observed that fractionation of the Cdt4 F20 main serine protease fraction occurred during reverse-phase HPLC using solution gradient A (0.05% trifluoroacetic acid, TFA) for 10 min to remove salt and the elution of serine protease fraction was done using a linear concentration gradient of buffer B (acetonitrile 66% in solution A). The fractionation was carried out at a flow rate of 2 mL/min, and monitoring of the elution was performed at 280 nm. Consequently, the main serine-protease fraction from Cdt4 F20 was separated into three new fractions, namely F201, F202, and F203, respectively. After pooling of fractions, lyophilization and final enzymatic confirmation, fraction F202 was subjected to structural, biochemical, and pharmacological tests.

During the fractionation, Figure 1A,C confirmed the molecular and biochemical differences between gyroxin and F202. Enzymatic and N-terminal sequencing confirmed that F202 was a serine protease, which exhibit differences from gyroxin. Therefore, F202 purified after several chromatography was designated as Cdtsp 2 that was subjected to several biochemical analysis. In the Supplementary Material Figure 1 (Figure S1) showed that Cdtsp 2 (F202) showed a Vmax of 3.33 µM/min and a Km value of 0.42. Two-dimensional SDS-PAGE confirmed the sample purity with a single molecular-weight band estimated at approximately 27.5 kDa, and a pI of approximately 4.5 and this protein Cdtsp 2 did not significantly reacted after assay for detection of carbohydrate structures in glycoprotein molecules using a Immun-Blot^®^ Kit for Glycoprotein Detection (Catalog Number 170-6490). Conventional SDS-Page showed that F202 had fibrinolytic activity, which revealed thrombin-like characteristics and showed the ability to degrade fibrinogen in three bands, α, β, and γ, with molecular weights of approximately 66 kDa, 55 kDa, and 45 kDa, respectively. After these steps of protein characterization, we performed a last chromatography to verify protein homogeneity using a last chromatography step on analytical C18 reverse phase HPLC. In this step, was used a discontinuous gradient concentration of buffer B, a same buffer used for all reverse phase HPLC chromatography, showing that Cdtsp 2 was 99% pure, and represented 5% of the total Cdt venom. The serine protease in F202 was obtained from the venom of *Crotalus durissus terrificus* (Cdtsp 2).

### 2.2. Structural Analysis

#### 2.2.1. Amino Acid Sequence

**Figure 2:**
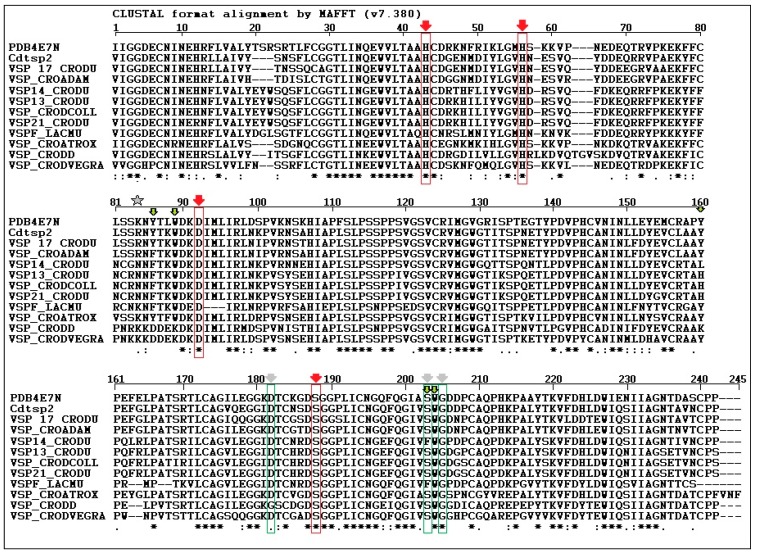
Amino acid alignment of the *Crotalus durissus terrificus* serine protease (Cdtsp 2) with the phylogenetic tree of snake venom serine proteases, including other gyroxin isoforms (venom snake protease VSP 17_CRODU—Swiss-Prot: B0FXM3.1, VSP13_CRODU—Swiss-Prot: B0FXM1.1, VSP14_CRODU—Swiss-Prot:B0FXM2.1, VSP_CRODCOLL—Swiss-Prot: A0A0S4FKT4.1, VSP 21 CRODU—Swiss-Prot:Q58G94, VSPF LACMU: Swiss-Prot:P33589, VSP CROATROX—Swiss-Prot: Q8QHK3.1 and VSP CRODVEGRA—Swiss-prot: A0A0U2UH64 and other snake venom serine proteases from *Crotalus adamanteus* (VSP_CROADAM—Swiss-Prot: J3S3W5.1 and VSP_CRODD—Swiss-Prot: Q2QA04.1). Residues marked in red correspond to the catalytic triad, and residues marked in green correspond to the substrate recognition sites. The star indicates the residue of Asparagine (N) which is crucial for the binding of carbohydrate residues and which is absent in Cdtsp 2. Arrows represents amino acid residues crucial to the biological and biochemical activity of serine protease from snake venoms.

The amino acid sequence of serine protease from *Crotalus durissus cascavella* venom was determined by Fonseca and colleagues [27] using Edman degradation, and it was confirmed by the Center for Research Support Facility (CEFAP) of the Institute of Biomedical Sciences (ICB/USP), therefore, this study was used as a guideline. The Cdtsp 2 protein sequence was used for comparison with other serine proteases identified in venom isolated from other snake species (Figure 2). Cdtsp2 has a nearly 90% sequence homology with the venom snake protease (VSP) [17] toxin and the serine protease isolated from *Crotalus adamantus*. When the Cdtsp 2 amino acid sequence was compared with that of other serine proteases from Cdt, its homology dropped to approximately 80%. Sequence analysis also revealed that Cdtsp 2 is a typical snake venom serine protease with all amino acids from the catalytic triad conserved (H43, D92, and S188).

#### 2.2.2. Circular Dichroism and Three-Dimensional Model of Cdtsp 2

**Figure 3:**
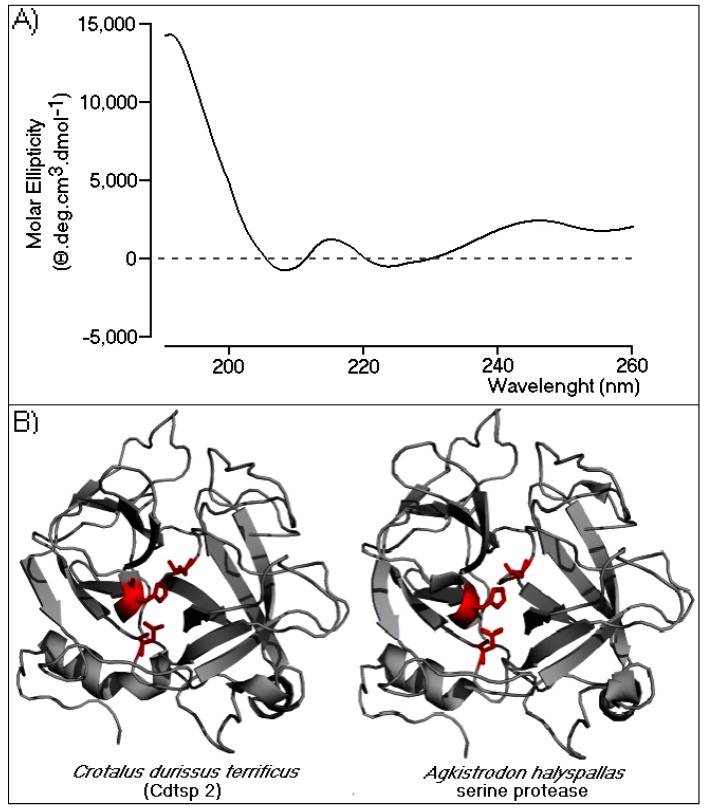
Secondary structural analysis and a structural model of Cdtsp 2. (**A**) The circular dichroism (CD) spectral profile of native Cdtsp 2. The data were acquired over the range of 185–280 nm. The CD spectra are expressed in θ machine units of millidegrees in molar ellipticity (θ cm^2^/dmols). (**B**) Three-dimensional model of Cdtsp 2. The structure is represented by gray ribbons. The amino acids from the catalytic triad are represented by red sticks.

Circular dichroism (CD) spectroscopy was performed to evaluate the secondary structure of Cdtsp 2. The profile revealed an α/β protein profile (Figure 3A), and data analysis showed that 60% of the Cdtsp2 enzyme structure was mostly in a random coil conformation, followed by β-sheet and α-helix structures (22% and 18%, respectively). To gain insight into the CD results, we built a three-dimensional model of Cdtsp 2, generated using the crystallographic structure of *Agkistrodon halys pallas* serine protease (PDB identifier: 4E7N). The model fit the data obtained by CD very well, which revealed a very high abundance of random coils, two β-sheet domains, and a few α helical structures (Figure 3B). As expected, in the model, the conserved catalytic triad (Ser, His, and Asp) was on the surface, rendering it very accessible to substrates (Figure 3B). Collectively, the CD and modeling data revealed that Cdtsp 2 had all the conventionally described structural elements characteristic of typical snake venom serine proteases.

### 2.3. Pharmacological Characterization of Cdtsp 2

#### 2.3.1. Edema Assay and Biochemical Determinations

**Figure 4:**
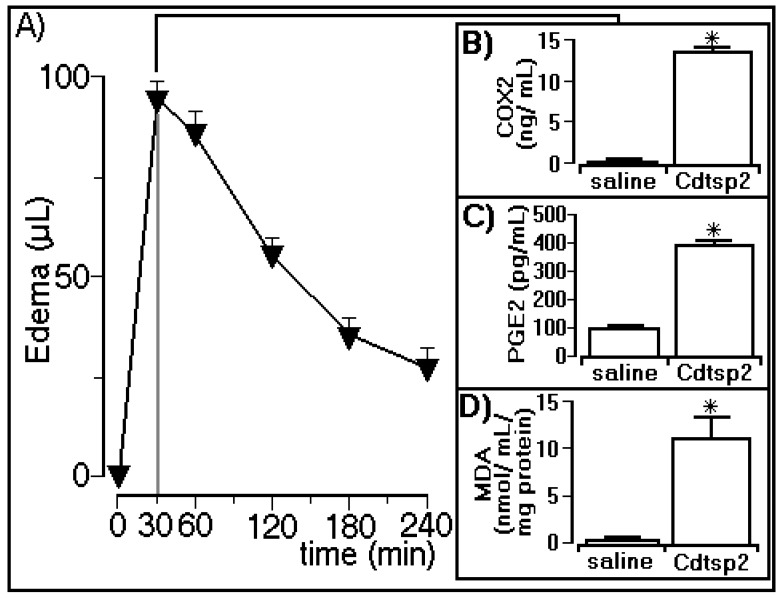
Paw edema induced by Cdtsp 2, and corresponding levels of cyclooxygenase 2 (COX-2) expression, and prostaglandin E2 (PGE2) and malondialdehyde (MDA) concentrations. (**A**) Volume of edema induced by Cdt serine protease. Plots (**B**–**D**) show the results for COX-2 expression, and for PGE2 and MDA concentrations, respectively. The results are expressed as means ± standard deviation (*n* = 5). Significant differences are marked with (*) (two-way ANOVA with Bonferroni as a posteriori test; *F* = 150.0 and *p* < 0.001).

The edema assay revealed that Cdtsp 2 induced a peak of 92 ± 6 µL in the animals (*n* = 5) 30 min after injection with Cdtsp 2 purified from Cdt venom (Figure 4A), and the biochemical analyses showed that Cdtsp 2 also induced an increase in COX-2 expression (Figure 4B). The biochemical analyses showed that Cdtsp 2 induced an increase in the plasma concentration of PGE2, which was four-fold higher than in animals receiving saline (Figure 4C). Therefore, the results of COX-2 and PGE2 analysis revealed that Cdtsp 2 can induce metabolism and the generation of prostaglandins (PGs). Therefore, the significant increase in MDA observed in animals treated with Cdtsp 2 compared with saline-treated animals (Figure 4D) was due to the lipid peroxidation of polyunsaturated fatty acids following an increase in oxidative stress. MDA is a widely used marker for the quantification of lipid peroxidation, which occurs when free radicals attack lipids, and an increase in MDA levels strongly suggests that Cdtsp 2 also induces an increase in oxidative stress.

#### 2.3.2. Edema Assay in the Presence of PLC, PKC, a cPLA2 Inhibitor, and a PAR1/2 Antagonist

**Figure 5:**
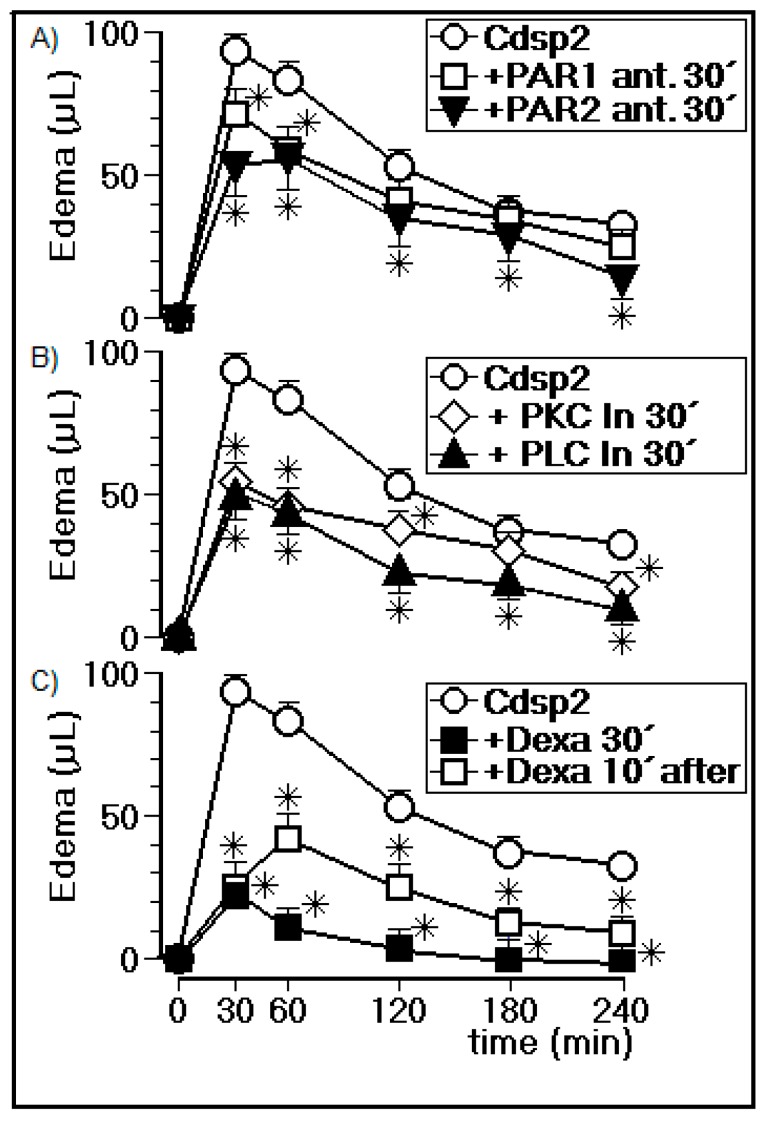
Paw edema assay in the presence of phospholipase C (PLC), protein kinase C (PKC), a cytosolic phospholipase A2 (cPLA2) inhibitor, and a protease-activated receptor 1 and 2 (PAR1/2) antagonist. (**A**) Edema induced by Cdtsp 2 in the presence of selective PAR1 and PAR2 antagonists, injected 30 min before the application of the protein. (**B**) Edema induced by Cdtsp 2 in the presence of selective inhibitors of PKC and PLC, injected 30 min before the application of the protein. (**C**) Evaluation of paw edema treated with dexamethasone (Dexa) injected 30 min before the application of Cdtsp 2, and 10 min after the application of the protein (10 μg of protein and 50 μg of selective inhibitors were used). The results are expressed as means ± standard deviation (*n* = 5). Significant differences are marked with (*) (two-way ANOVA with Bonferroni as a posteriori test; *F* = 150.0 and *p* < 0.001).

In Figure 5A, we show the effects of PAR1 and PAR2 antagonists injected into animals (*n* = 5) 30 min before injection with Cdtsp 2. Figure 5A reveals that the PAR1 inhibitor reduced the peak volume of edema from 92 ± 6 μL (*n* = 5) to 71 ± 8 μL (*n* = 5). In the case of the PAR2 inhibitors, the peak volume of dropped to 52 ± 9 μL (*n* = 5) 30 min after injection withCdtsp 2. In addition, Figure 5A shows that the volumes of edema induced by Cdtsp 2 in animals treated with the PAR1 and PAR2 antagonists were also reduced across all time points. The PLC and PKC inhibitor results are shown in Figure 5B, revealing that the PKC enzyme inhibitor significantly reduced the peak volume of edema from 92 ± 6 μL (*n* = 5 animals) to 58 ± 9 μL (*n* = 5 animals), while the PLC inhibitor reduced peak volume of edema to 46 ± 9 μL (*n* = 5 animals) 30 min after injection with Cdtsp 2. Figure 5B also shows that the PLC inhibitor practically abolished the proinflammatory effects induced by Cdtsp 2 throughout the edema assay, while the PKC inhibitor reduced the Cdtsp 2-induced edema at the 30 min and 60 min time points. Figure 5C shows the effect of dexamethasone (Dexa) on the edema induced by Cdtsp 2. The peak volume of edema induced by Cdtsp 2 in animals treated with Dexa was 25 ± 8 μL (*n* = 5). Lastly, Figure 5C also shows that animals treated with Dexa 30 min before injection with Cdtsp 2 abolished the edema induced by the Cdtsp 2 serine protease. Figure 5C further shows the anti-inflammatory effect of Dexa on the Cdtsp 2-induced edema. The drug was applied to animals 10 min after injection with the serine protease. Therefore, these data clearly show that Cdtsp 2 induces significant proinflammatory effects, and is mediated by arachidonic acid mobilization.

## 3. Discussion

In this study, we performed structural analysis involving the primary, secondary, and tertiary structures through analysis of the protein sequence, CD spectroscopy, and the building of a three-dimensional model of Cdtsp 2 (Figure 2 and Figure 3). Together, our results revealed that Cdtsp 2 has a high degree of structural similarity to other snake venom serine proteases, including the presence of a hydrophobic cavity on its surface, and the conservation of amino acid residues involved in the activity of snake venom serine protease, such as H43, H56, D92, and S188 (Figure 2) [11,28]. These conserved amino acid residues are located between the N-terminal and C-terminal regions, which features a hydrophobic cavity typical of major serine proteases, containing the catalytic triad of residues [2,5,16,28] (Figure 1 and Figure 2B). A study on serine proteases isolated from *Lachesis muta* revealed that the residues, F86, W89, Y160, F203, and W204, form an essential hydrophobic cavity involved in binding fibrinopeptide A and chromogenic substrates [22]. Our results show that Cdtsp 2 also includes S86, W89, Y160, S203, and W204 residues, which are structurally equivalent to those found in the *L. muta* serine protease and in human thrombin [22].

A study by Patiño and coworkers [3] focused on two trypsin-like serine proteases with a high affinity for the BAPNA substrate, isolated from *Crotalus durissus cumanensis* venom. These serine proteases also presented fibrinolytic activity and other enzymatic activities typically found in other serine proteases isolated from snake venom. In addition, these trypsin-like serine proteases induced moderate edema, as also observed with Cdtsp 2. However, remarkable volumes of edema were only observed in concentrations higher than 5 μg per animal (5 mg/mL) of Cdtsp 2, which is less intense than volumes of edema promoted by Cdt sPLA2 [29]. Cdtsp 2 induces substantial plasma coagulation, and mice passed away when more than 20 μg per animal (20 mg/mL) of Cdtsp 2 was used. Therefore, we decided to use 10 μg (10 mg/mL) of Cdtsp 2 in our assays.

In general, the inflammatory process is based on the role of AA and its mobilization in conjunction with COX-2 activity. AA is a component of membrane phospholipids that may be released in a one-step process following PLA2 action, or in a two-step process following the actions of PKC and DAG lipase. However, other studies revealed that some kinases play a key role in cPLA2 activation via enzyme phosphorylation. COX and 5-lipoxygenase metabolize AA, resulting in the syntheses of PG and leukotriene, respectively. During the inflammatory process, this aforementioned two-step process results in the oxygenation of AA. However, COX transforms AA into endoperoxides, which are subsequently used to synthesize prostaglandins, PGIs, or TXs. Drugs such as aspirin and indomethacin inhibit COX activity, thereby blocking the syntheses of prostaglandin and TX [26]. The COX-2 enzyme catalyzes the conversion of AA into various prostaglandins, such as PGE2, which are associated with an increase in the synthesis of proinflammatory cytokines, tumor necrosis factor alpha (TNF-α), and interleukin 1 beta (IL-1β). Furthermore, these mediators are formed following the hydrolytic action of PLA2 on cell membrane phospholipids, resulting in the release of AA, as well as the production of other metabolites such as 8-isoprostanes, which can undergo nonenzymatic peroxidation via intermediate reactive oxygen species [14,18,29,30,31,32,33,34,35].

Our results suggest that the lipid peroxidation and AA metabolism induced by Cdtsp 2 during acute edema are interrelated. A common signaling pathway also triggers these enzymatic and metabolic activities. According to some studies, AA metabolism itself may be a significant source of reactive oxygen species, such as those induced by sPLA2 and PLC. Its metabolites, DAG and IP_3_, are involved, together with PKC, in the molecular simulation pathway for the mobilization of AA by Cdtsp 2. Furthermore, cPLA2 displays an essential activity in inflammation induced by Cdtsp 2, which was shown by the inhibition of edema in the presence of dexamethasone [36]. Our results clearly demonstrate a molecular link between PAR1 and PAR2 as a key feature of the edema induced by this serine protease. PAR1 and PAR2 are involved in the modulation of the inflammatory process, and are constitutively expressed in mast cells, while PAR3 and PAR4 are involved in PAR4 activation and the modulation of platelet activity [37]. Furthermore, PAR1 is generally recognized by several coagulation factors other than thrombin [38]. Furthermore, the acute edema assays suggested that Cdtsp 2 is a proinflammatory agent. Therefore, the choice of PAR1 and PAR2 inhibitors was based on an extensive review of the literature [39]. 

In the case of human thrombin, PARs are linked with the activation of PKC, as well as that of PLC and the production of its main metabolites, IP_3_ and DAG [40,41]. In summary, we observed that, under our experimental conditions, acute edema induced by Cdtsp 2 involves a G-protein signaling pathway coupled to PAR-type receptors. A previous study showed that the administration of specific concentrations of PAR agonists may not only decrease inflammation, but also induce hyperalgesia [42]. Lastly, our results clearly suggest that edema induced by Cdtsp 2 leads to increased oxidative stress, as shown by the increase in levels of MDA. Several studies revealed an association between increased AA metabolism and high levels of cellular oxidative stress due to the development of reactive oxygen species such as hydrogen peroxide [43,44]. Another study suggested that this cellular oxidative stress may be the result of an increase in nicotinamide adenine dicleotide phosphate (NADPH) oxidase activity, a key enzyme involved in cellular oxidative stress [45]. In summary, the edema induced by Cdtsp 2 mobilizes AA and leads to its metabolism, thereby potentially increasing cellular oxidative stress.

Despite the structural similarity of Cdtsp 2 to other snake venom serine proteases, established by the presence of a hydrophobic cavity on its surface and the conservation of amino acid residues involved in its activity, each protein is unique. As such, it is not possible to conclude that all SVSPs are involved in the activation of proinflammatory pathways, despite our data revealing Cdtsp 2 as the first serine protease with this activity.

## 4. Materials and Methods

### 4.1. Cdtsp 2 Purification

The venom from *Crotalus durissus terrificus* (Cdt) was kindly donated by the Butantan Institute (São Paulo, Brazil). The solvents, chemicals, and reagents used for protein purification and characterization (HPLC grade or higher) were acquired from Sigma-Aldrich Chemicals (Spruce St., St. Louis, MO, USA), Merck (Whitehouse Station, NJ, USA), and Bio-Rad (Hercules, CA, USA). Venom purification was carried out in a Superdex G75 chromatographic column, pre-packed on an HPLC column (1.6 × 60 cm), previously equilibrated with two column volumes of ammonium bicarbonate buffer (0.1 M, pH 7.9) with an isocratic flow maintained at 0.2 mL/min. The fractions were collected at 2-min intervals, and monitoring was performed at 280 nm absorbance. Fractions were tested for serine-protease activity and levels of phospholipase A2 at 405 nm and 425 nm, respectively, as well as being monitored on the basis of plasma coagulation time. Fractions with activities were then grouped. The sample purified from total venom was repurified on a DEAE-5PW (Supelco, 2033 Westport Center Dr, St. Louis, MO, USA) ion exchange column. Fractionation was performed on a column previously equilibrated with 0.05 M ammonium bicarbonate buffer, pH 7.9, and the fractionation of samples was carried out in a three-step protocol. C4 reverse-phase HPLC was done using solution gradient A (0.05% TFA) for 10 min to remove salt, followed by a stepwise gradient with 100% solution B (acetonitrile 66% in solution A). Fractionation was carried out at a flow rate of 2 mL/min, and monitoring of the elution was performed at 280 nm. Purification of F202 (Cdtsp 2) was completed using C18 reverse-phase chromatography.

### 4.2. Coagulation Activity

The evaluation of thrombin clotting time was performed through a turbidimetry test in an appropriate photometer (Human Clot junior). Thrombin catalyzes the conversion of fibrinogen to fibrin at the end of the coagulation cascade, resulting in an increase in sample turbidity, which was detected by the apparatus. This detection was started manually after the reagent was added to the solution. The time (seconds) between the mixing of the reagents and the final turbidity was recorded. The following treatments were used: 45 μL of commercial human plasma, 45 μL of a stock solution of gyroxin (hemostatic thrombin time), or 45 μL of Cdtsp 2 (hemostatic thrombin time), and 10 μL of saline. The turbidity (at a wavelength of 400 nm) was recorded, and the data were subsequently subjected to an analysis of variance (*p* < 0.005). Bovine thrombin was used as a positive control [46].

### 4.3. Primary Structure Determination

This novel serine protease was purified from Cdt snake venom, following the method described by Fonseca and coworkers [27]. The amino acid sequence and characterization of the new serine protease was performed using tryptic digestion and the purification of peptide fragments. The determination of its primary structure was performed using other serine proteases isolated from Cdt as primary sequences for the overlapping of tryptic peptides in Edman degradation. The alignment sequence was made using the Clustal program. The primary structure of Cdtsp 2 was also confirmed through protein sequencing (mass spectrometry) undertaken by the Research Support Facility Center (CEFAP) at the Biomedical Sciences Institute (ICB) of the University of São Paulo (USP).

### 4.4. Circular Dichroism Spectroscopy

Polarized light was used in the distal ultraviolet (UV) range (from 190 nm to 260 nm). This technique allows the evaluation of the structural integrity of proteins, conformational changes, and processes of denaturation (unfolding) and renaturation (folding), allowing the estimation of the elemental composition in the secondary structure of this macromolecule. Circular dichroism analysis was performed with 0.02 mg/mL Cdtsp 2 and 400 μL of Tris pH (7.4) buffer. The analysis was subjected to eight convolutions, and the data were treated by the Spectra manager.

### 4.5. Structural Homology Modeling

Models of the tertiary structure were obtained using coordinates of serine proteases present in the protein data bank (PDB), which presented greater similarities than those identified in Cdt. For the construction of the models, the Swiss-Model package [46] was used to the nearest molecule with previously deposited molecular coordinates. The serine protease of *Agkistrodon halys* (PDB identifier: 4E7N) had the highest homology; thus, the Cdtsp 2 model was constructed based on these coordinates. The structural model of Cdtsp 2 was generated using Uniprot with energy-minimization parameters [47]. Molecular representations were generated using the PyMOL software, (120 West 45th Street 17th Floor New York, NY, USA) [48].

### 4.6. Enzymatic Activity 

The protocol designed by Prasa et al. [49] was used to perform the enzymatic assay with detection at a wavelength of 405 nm. The reagents used included 50 mM Tris-HCl buffer (pH 7.4), 100 mM NaCl, and 2 mg/mL Nα-benzoyl-l-arginine 4-nitroanilide (BAPNA), a suitable trypsin substrate. Furthermore, three assays were done including the substrate only, 1 mg/mL protein incubated with the substrate, and 1 mg/mL gyroxin incubated with the substrate. The reagents were then pipetted onto a microplate reader incubated at 37 °C. The same protocol was followed for the determination of phospholipase A2 activity at a wavelength of 425 nm, using 50 mM Tris-HCl buffer (pH 7.8), 100 mM NaCl, and the 4-nitro-3-octanoyloxybenzoic (NOB) protein as a substrate (2 mg/mL). The readings were performed on a UV-VIS SpectraMax 190 (Molecular Devices, LLC., 3860 N First Street, San Jose, CA, USA).

### 4.7. Quantification of Enzymes

Animals were used for the enzyme quantification carried out with commercial kits. Blood was collected from mice with heparin, before being centrifuged. The plasma was separated and stored at −80 °C for use in the COX-2 and MDA assays (Abcam, Cambridge, MA, USA). The right gastrocnemius muscle was collected and stored at −80 °C for PGE2 quantification.

#### 4.7.1. Evaluation of COX-2 Levels

COX-2 levels were analyzed using an enzyme-linked immunosorbent assay (ELISA) kit (ab210574; Abcam, Cambridge, MA, USA), according to the manufacturer’s guidelines.

#### 4.7.2. Prostaglandin E2 Quantification

PGE2 levels were determined using an ELISA kit (ab133021; Abcam, Cambridge, MA, USA), according to the manufacturer’s guidelines.

#### 4.7.3. Lipid Peroxidation Determination

The extent of lipid peroxidation was determined via the monitoring of MDA levels using an ELISA kit (ab118970; Abcam, Cambridge, MA, USA), according to the manufacturer’s guidelines.

### 4.8. Evaluation of Paw Edema

Female Swiss mice (20–25 g, *n* = 5) were obtained from the Multidisciplinary Centre for Biological Research (CEMIB) of the State University of Campinas (UNICAMP). The animals were maintained under standard conditions (25 °C; 12-h light/dark cycle) with food and water available ad libitum. In vivo experimental models were performed to evaluate the inhibition of acute inflammation induced by purified Cdtsp 2, using randomly chosen mice (*Mus musculus*). These assays were performed only after an in vitro investigation of the inhibition or interaction of the compounds with the enzymes used in this study. Animals were treated with 50 μL (0.5 μg/μL per animal; 10 μg per animal) of compound, administered through peritoneal or intravenous injection. A saline solution (0.9% NaCl) was used as the negative control. In total, 14 groups were included: saline, Cdtsp 2, Cdtsp 2 + PKC, Cdtsp 2 + PLC, Cdtsp 2 + PAR1 antagonist, Cdtsp 2 + PAR2 antagonist, PKC, PLC, PAR1 antagonist, PAR2 antagonist, two dexamethasone groups, and two Cdtsp 2 groups + dexamethasone. The monitoring of edema volume was performed using a digital plethysmometer for approximately four hours. After the tests, the mice were anesthetized and sacrificed through a cervical dislocation. The in vivo experiments were performed according to the institutional rules, and were approved by the ethics committee of UNESP (number 014-CEUA, 30 August 2016.

#### 4.8.1. PAR1 Antagonist (ML161) and PAR2 Antagonist (SML 1039)

A total of 100 μL (50 μg) of PAR1 antagonist, ML161 (Sigma; half maximal inhibitory concentration (IC_50_) = 0.026 μM, dissolved in 0.5% dimethyl sulfoxide (DMSO)), was injected intraperitoneally 30 min before the application of 20 μL (10 µg per animal) of the Cdtsp 2 protein to the right paw. Additionally, 100 μL (50 μg per animal) of PAR2 antagonist, SML 1039 (Sigma; IC_50_ = 1.2 mM, dissolved in saline), was injected intraperitoneally 30 min before the application of 20 μL (10 μg per animal) of the Cdtsp 2 protein to the right paw.

#### 4.8.2. PLC Inhibition by U73122

A total of 50 μL (20 μg) of U73122 (Tocris), a PLC inhibitor (30 mg/kg, dissolved in 0.5% DMSO), was injected intravenously 30 min before the application of 20 μL (10 µg per animal) of the Cdtsp 2 protein to the right paw.

#### 4.8.3. PKC Inhibition by GF109203X

A total of 50 μL (20 μL) of the PKC inhibitor, GF109203X (Tocris; 30 mg/kg, dissolved in 0.5% DMSO), was injected into the right paw 30 min before the application of 20 μL (10 μg) of the Cdtsp 2 proteinto the right paw.

#### 4.8.4. Dexamethasone Treatment

A total of 100 μL (50 μg per animal) of the anti-inflammatory corticosteroid, dexamethasone, for veterinary use (Corvet; 0.5 mL/kg per animal) was injected intraperitoneally into the right paw 10 min after injection of the Cdtsp 2 protein. In another group, the same procedure was followed, with the application of dexamethasone occurring 30 min before the application of 20 μL (10 μg) of the Cdtsp 2 protein to the right paw.

### 4.9. Statistical Analysis

Data are expressed as means ± standard deviation. The results were analyzed using one-way or two-way analysis of variance (ANOVA), followed by the Bonferroni test and *t*-test, with statistical variance determined for *p* < 0.05.

## 5. Conclusions

**Figure 6:**
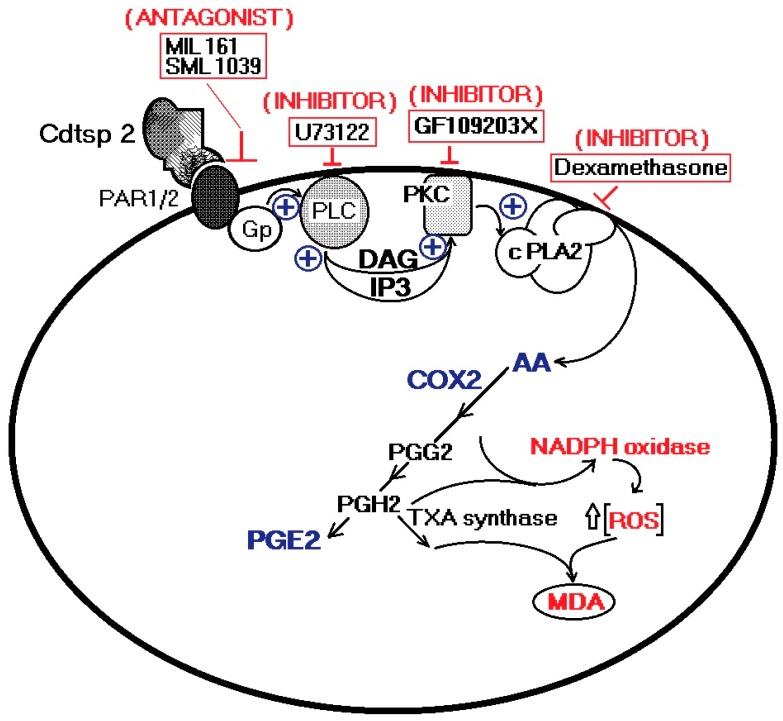
Hypothetical pathway involved in the proinflammatory effects of Cdtsp 2 on mouse tissue. Cdtsp 2 induces edema via an enzymatic cleavage of PAR1 and PAR2 coupled with a G-protein, subsequently activating PLC and PKC. Consequently, an increase in cPLA2 activity is triggered by an increase in AA metabolism and its interplay with oxidative stress.

Cdtsp 2 is a novel serine protease, isolated from *Crotalus durissus terrificus* venom, which presents enzymatic activity similar to that of trypsin, with no significant signs of esterase activity. The results strongly suggest that the number of serine proteases present in Cdt venom is higher than that currently known for *Crotalus* ssp. venom in the literature. Our data show that Cdtsp 2 possesses structural aspects intrinsic to other SVSPs, and presents significant edematogenic activity, involving the indirect mobilization of arachidonic acid, and its consequent mobilization by cPLA2 and COX-2, in addition to increasing oxidative stress (Figure 6). Moreover, the role of serine proteases in modulating blood homeostasis could also feature an essential inflammatory activity that is not yet fully understood.

## Supplementary Materials

Supplementary materials can be found at http://www.mdpi.com/1422-0067/19/8/2405/s1, Figure S1: Enzymatic assay of Cdtsp 2 (V0 µMol/min vs. BAPNA (mM)). Figure S2: In the attached figure, we present a set of results compiled showing the fractionation of the whole crotoxin and the CA (acid component, crotoxin A, crotapotin, and Crpt), CB (basic component, crotoxin B, secretory phospholipase A2, and sPLA2), and a new group of proteases that was eluted with fraction F20.

## References

[1] De Oliveira D.G.L., Murakami M.T., Cintra A.C.O., Franco J.J., Sampaio S.V., Arni R.K. (2009). Functional and structural analysis of two fibrinogen-activating enzymes isolated from the venoms of *Crotalus durissus terrificus* and Crotalus durissus collilineatus. Acta Biochim. Biophys. Sin. (Shanghai).

[2] Boldrini-França J., Rodrigues R.S., Santos-Silva L.K., de Souza D.L.N., Gomes M.S.R., Cologna C.T., de Pauw E., Quinton L., Henrique-Silva F., de Melo Rodrigues V. (2015). Expression of a new serine protease from *Crotalus durissus collilineatus* venom in *Pichia pastoris* and functional comparison with the native enzyme. Appl. Microbiol. Biotechnol..

[3] Patiño A.C., Pereañez J.A., Gutiérrez J.M., Rucavado A. (2013). Biochemical and biological characterization of two serine proteinases from Colombian *Crotalus durissus cumanensis* snake venom. Toxicon.

[4] Toyama M.H., Toyama D.D.O., Passero L.F.D., Laurenti M.D., Corbett C.E., Tomokane T.Y., Fonseca F.V., Antunes E., Joazeiro P.P., Beriam L.O. (2006). Isolation of a new l-amino acid oxidase from *Crotalus durissus cascavella* venom. Toxicon.

[5] Yonamine C.M., Prieto-da-Silva A.R., Magalhães G.S., Rádis-Baptista G., Morganti L., Ambiel F.C., Chura-Chambi R.M., Yamane T., Camillo M.A. (2009). Cloning of serine protease cDNAs from *Crotalus durissus terrificus* venom gland and expression of a functional Gyroxin homologue in COS-7 cells. Toxicon.

[6] Menaldo D.L., Bernardes C.P., Pereira J.C., Silveira D.S.C., Mamede C.C., Stanziola L., Oliveira F.D., Pereira-Crott L.S., Faccioli L.H., Sampaio S.V. (2013). Effects of two serine proteases from *Bothrops pirajai* snake venom on the complement system and the inflammatory response. Int. Immunopharmacol..

[7] Da-Silva-Freitas D., Boldrini-França J., Arantes E.C. (2015). PEGylation: A successful approach to improve the biopharmaceutical potential of snake venom thrombin-like serine protease. Protein Pept. Lett..

[8] Menaldo D.L., Bernardes C.P., Zoccal K.F., Jacob-Ferreira A.L., Costa T.R., Del Lama M.P., Naal R.M., Frantz F.G., Faccioli L.H., Sampaio S.V. (2017). Immune cells and mediators involved in the inflammatory responses induced by a P-I metalloprotease and a phospholipase A2 from Bothrops atrox venom. Mol. Immunol..

[9] Mamede C.C.N., de Sousa B.B., da Cunha Pereira D.F., Matias M.S., de Queiroz M.R., de Morais N.C.G., Vieira S.A.P.B., Stanziola L., Oliveira F. (2016). Comparative analysis of local effects caused by Bothrops alternatus and Bothrops moojeni snake venoms: Enzymatic contributions and inflammatory modulations. Toxicon.

[10] Fox J.W., Serrano S.M. (2008). Insights into and speculations about snake venom metalloproteinase (SVMP) synthesis, folding and disulfide bond formation and their contribution to venom complexity. FEBS J..

[11] Zychar B.C., Dale C.S., Demarchi D.S., Gonçalves L.R.C. (2010). Contribution of metalloproteases, serine proteases and phospholipases A2 to the inflammatory reaction induced by *Bothrops jararaca* crude venom in mice. Toxicon.

[12] Leslie C.C. (2015). Cytosolic phospholipase A(2): Physiological function and role in disease. J. Lipid. Res..

[13] Burke J.E., Dennis E.A. (2009). Phospholipase A(2) structure/function, mechanism, and signaling. J. Lipid Res..

[14] Lindgren C.A., Newman Z.L., Morford J.J., Ryan S.B., Battani K.A., Su Z. (2013). Cyclooxygenase-2, prostaglandin E2 glycerol ester and nitric oxide are involved in muscarine-induced presynaptic enhancement at the vertebrate neuromuscular junction. J. Physiol..

[15] Norris P.C., Gosselin D., Reichart D., Glass C.K., Dennis E.A. (2014). Phospholipase A(2) regulates eicosanoid class switching during inflammasome activation. Proc. Natl. Acad. Sci. USA.

[16] Mouchlis V.D., Dennis E.A. (2016). Membrane and Inhibitor Interactions of Intracellular Phospholipases A(2). Adv. Biol. Regul..

[17] Sun G.Y., Chuang D.Y., Zong Y., Jiang J., Lee J.C.M., Gu Z., Simonyi A. (2014). Role of cytosolic phospholipase A(2) in oxidative and inflammatory signaling pathways in different cell types in the central nervous system. Mol. Neurobiol..

[18] Farooqui A.A., Horrocks L.A. (2005). Signaling and interplay mediated by phospholipases A2, C, and D in LA-N-1 cell nuclei. Reprod. Nutr. Dev..

[19] Cosentino-Gomes D., Rocco-Machado N., Meyer-Fernandes J.R. (2012). Cell Signaling through Protein Kinase C Oxidation and Activation. Int. J. Mol. Sci..

[20] Cavada B.S., Castellón R.E.R., Vasconcelos G.G., Rocha B.A.M., Bezerra G.A., Debray H., Delatorre P., Nagano C.S., Toyama M., Pinto V.P. (2005). Crystallization and preliminary X-ray diffraction analysis of a new chitin-binding protein from *Parkia platycephala* seeds. Acta Crystallogr. Sect. F Struct. Biol. Cryst. Commun..

[21] Newton A.C. (2010). Protein kinase C: Poised to signal. Am. J. Physiol. Endocrinol. Metab..

[22] Grimsey N., Soto A.G., Trejo J. (2011). Regulation of Protease-activated Receptor Signaling by Posttranslational Modifications. IUBMB Life.

[23] Covic L., Gresser A.L., Talavera J., Swift S., Kuliopulos A. (2002). Activation and inhibition of G protein-coupled receptors by cell-penetrating membrane-tethered peptides. Proc. Natl. Acad. Sci. USA.

[24] Farooqui A.A., Horrocks L.A. (2006). Phospholipase A_2_-Generated Lipid Mediators in the Brain: The Good, the Bad, and the Ugly. Neuroscientist.

[25] Liu C., Liu B., Liu L., Zhang E.-L., Sun B., Xu G., Chen J., Gao Y.Q. (2018). Arachidonic Acid Metabolism Pathway Is Not Only Dominant in Metabolic Modulation but Associated With Phenotypic Variation After Acute Hypoxia Exposure. Front. Physiol..

[26] Sun G.Y., He Y., Chuang D.Y., Lee J.C., Gu Z., Simonyi A., Sun A.Y. (2012). Integrating cytosolic phospholipase A2 with oxidative/nitrosative signaling pathways in neurons: A novel therapeutic strategy for AD. Mol. Neurobiol..

[27] Fonseca F.V., Antunes E., Morganti R.P., Monteiro H.S., Martins A.M., Toyama D.O., Marangoni S., Toyama M.H. (2006). Characterization of a new platelet aggregating factor from crotoxin *Crotalus durissus cascavella* venom. Protein J..

[28] Hamilton J.R., Trejo J. (2017). Challenges and Opportunities in Protease-Activated Receptor Drug Development. Ann. Rev. Pharmacol. Toxicol..

[29] Costa Jde O., Fonseca K.C., Garrote-Filho M.S., Cunha C.C., de Freitas M.V., Silva H.S., Araújo R.B., Penha-Silva N., de Oliveira F. (2010). Structural and functional comparison of proteolytic enzymes from plant latex and snake venoms. Biochimie.

[30] Castro H.C., Silva D.M., Craik C., Zingali R.B. (2001). Structural features of a snake venom thrombin-like enzyme: Thrombin and trypsin on a single catalytic platform?. Biochim. Biophys. Acta Protein Struct. Mol. Enzymol..

[31] Ricciotti E., FitzGerald G.A. (2011). Prostaglandins and Inflammation. Arterioscler. Thromb. Vasc. Biol..

[32] Maroun R.C., Serrano S.M.T. (2003). Identification of the substrate-binding exosites of two snake venom serine proteinases: Molecular basis for the partition of two essential functions of thrombin. J. Mol. Recognit..

[33] Upadhyay A.K., Singh A., Mukherjee K.J., Panda A.K. (2014). Refolding and purification of recombinant L-asparaginase from inclusion bodies of E. coli into active tetrameric protein. Front. Microbiol..

[34] Goettig P. (2016). Effects of Glycosylation on the Enzymatic Activity and Mechanisms of Proteases. Int. J. Mol. Sci..

[35] Coorssen J.R. (1996). Phospholipase activation and secretion: Evidence that PLA2, PLC, and PLD are not essential to exocytosis. Am. J. Physiol. Physiol..

[36] Gijón M.A., Leslie C.C. (1999). Regulation of arachidonic acid release and cytosolic Phospholipase A2 activation. J. Leukoc. Biol..

[37] Balsinde J., Balboa M.A., Dennis E.A. (2000). Identification of a Third Pathway for Arachidonic Acid Mobilization and Prostaglandin Production in Activated P388D1 Macrophage-like Cells. J. Biol. Chem..

[38] Touqui L., Alaoui-El-Azher M. (2001). Mammalian Secreted Phospholipases A2 and Their Pathophysiolo-gical Significance in Inflammatory Diseases. Curr. Mol. Med..

[39] Xu J., Weng Y.I., Simonyi A., Krugh B.W., Liao Z., Weisman G.A., Sun G.Y. (2002). Role of PKC and MAPK in cytosolic PLA2 phosphorylation and arachadonic acid release in primary murine astrocytes. J. Neurochem..

[40] Balboa M.A., Balsinde J. (2006). Oxidative stress and arachidonic acid mobilization. Biochim. Biophys. Acta Mol. Cell Biol. Lipids..

[41] Soubhye J., van Antwerpen P., Dufrasne F. (2018). Targeting Cytosolic Phospholipase A2α for Novel Anti-Inflammatory Agents. Curr. Med. Chem..

[42] Yonamine C.M., Kondo M.Y., Juliano M.A., Icimoto M.Y., Baptista G.R., Yamane T., Oliveira V., Juliano L., Lapa A.J., Lima-Landman M.T. (2012). Kinetic characterization of gyroxin, a serine protease from *Crotalus durissus terrificus* venom. Biochimie.

[43] Dale C., Vergnolle N. (2008). Protease Signaling to G Protein-Coupled Receptors: Implications for Inflammation and Pain. J. Recept. Signal Transduct. Res..

[44] Houle S., Papez M.D., Ferazzini M., Hollenberg M.D., Vergnolle N. (2005). Neutrophils and the kallikrein-kinin system in proteinase-activated receptor 4-mediated inflammation in rodents. Br. J. Pharmacol..

[45] Park K.W., Jin B.K. (2008). Thrombin-induced oxidative stress contributes to the death of hippocampal neurons: Role of neuronal NADPH oxidase. J. Neurosci. Res..

[46] Maldonado E., Sunagar K., Almeida D., Vasconcelos V., Antunes A. (2014). IMPACT_S: Integrated Multiprogram Platform to Analyze and Combine Tests of Selection. PLoS ONE.

[47] (2016). The UniProt Consortium. UniProt: The universal protein knowledgebase. Nucleic Acids Res..

[48] Lua R.C., Lichtarge O. (2010). PyETV: A PyMOL evolutionary trace viewer to analyze functional site predictions in protein complexes. Bioinformatics.

[49] Prasa D., Svendsen L., Stürzebecher J. (1997). Inhibition of thrombin generation in plasma by inhibitors of factor Xa. Thromb. Haemost..

