# Supplementary material for: Edema Induced by a Crotalus durissus terrificus Venom Serine Protease (Cdtsp 2) Involves the PAR Pathway and PKC and PLC Activation
Source: Int J Mol Sci. 2018 Aug 15;19(8):2405. doi: 10.3390/ijms19082405 (PMC6121655; doi:10.3390/ijms19082405)
Supplement: Supplementary file 1 [file ijms-19-02405-s001.pdf]

Supplementary materials

Edema induced by a *Crotalus durissus terrificus* venom serine protease (Cdtsp 2) involves the PAR pathway and PKC and PLC activation.

Caroline R.C. Costa[1], Mariana N. Belchor[1], Caroline F. B. Rodrigues[3], Daniela de O. Toyama[1], Marcos A. de Oliveira2, Danielle P. Novaes[1]and Marcos H. Toyama[1,*].

Supplementary Results

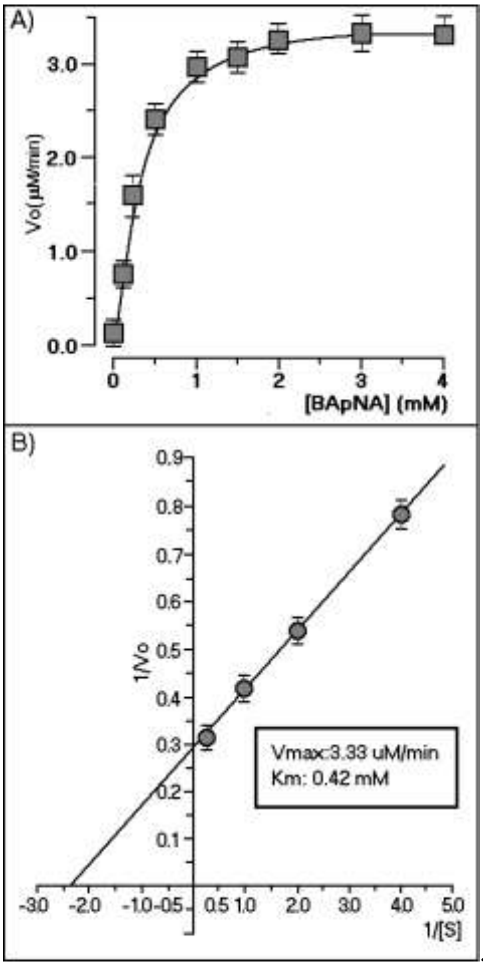

Figure S1. Graphics of the Cdtsp2 enzymatic assay. (**A)** Enzymatic assay of Cdtsp2 (V_0 (μMol/min) vs. Nα-benzoyl-l-arginine 4-nitroanilide (BAPNA;mM)). (**B)** V_{max} and K_m values were obtained from a Lineweaver–Burk plot. The protocol designed by Prasa et al. [49] was used to perform this test. The reagents used were 50 mM Tris-HCl buffer (pH 7.4) 100 mM NaCl, and a thrombin generation chromatogenic substrate (thrombin-like; 1mg/ml). The three points in the graph represent a control group with only the substrate, Cdtsp2 (1mg/ml) incubated with substrate, and gyroxin (1mg/ml) incubated with substrate, in a microplate reader incubated at 37 °C. The readings were determined on a SPECTRA MAX (Molecular Devices, CA) at a wavelength of 405nm.

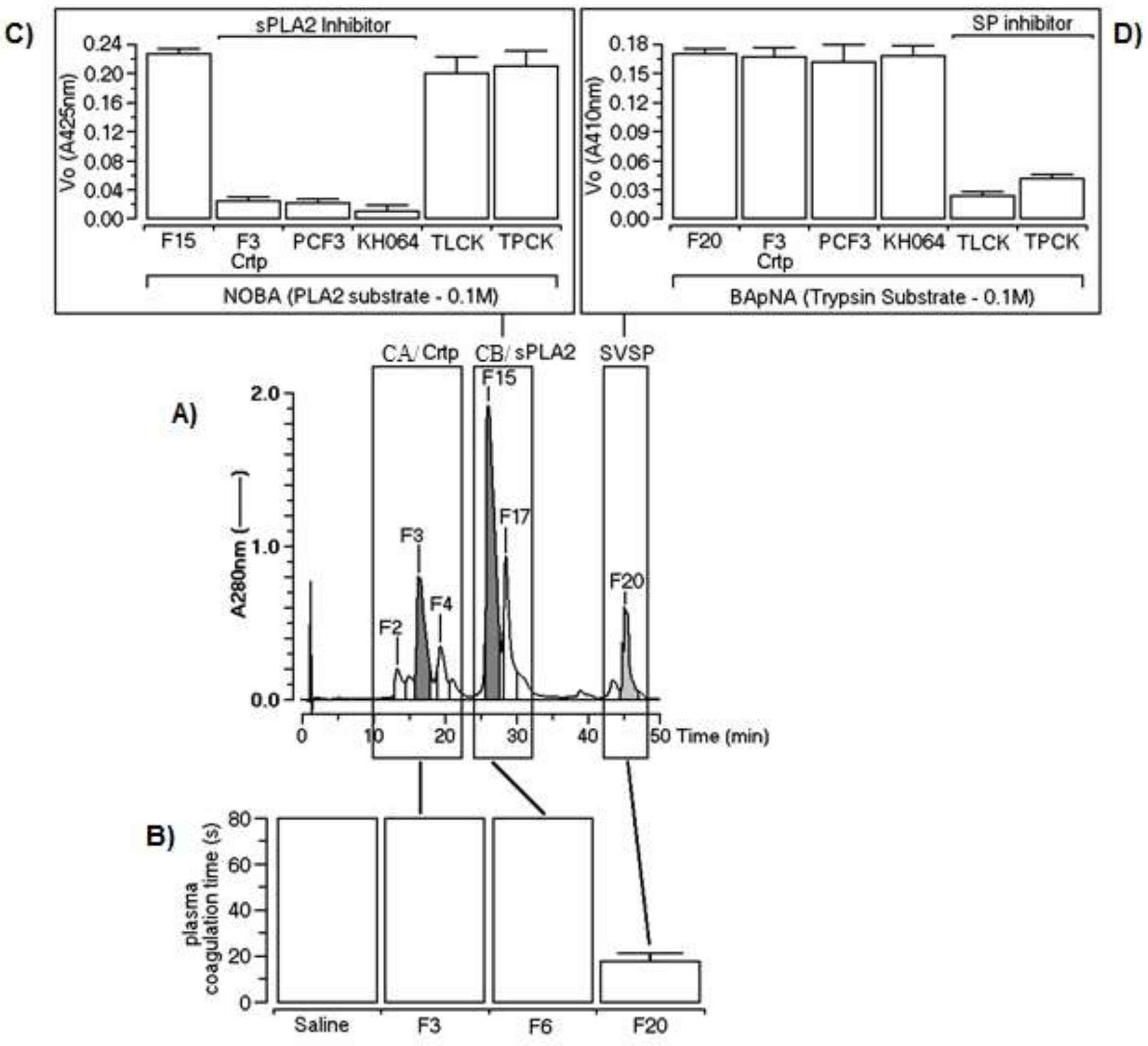

Figure S2. In the attached figure, we present a set of results compiled showing the fractionation. (**A**) Venom purification using a Superdex G75 chromatographic column pre-packed on an HPLC column (1.6 x 60 cm) previously equilibrated with two column volumes of ammonium bicarbonate buffer (0.1M, pH 7.9). The isocratic flow was maintained at 0.2 mL/min for the whole crotoxin fraction, as well as for the fractionation of CA (acid component, crotoxin A; crotapotin (Crpt)), CB (basic component, crotoxin B; secretory phospholipase A2 (sPLA2)), and a new group of proteases that were eluted with fraction F20. After a period of chromatography, F20 produced the serine protease, F202, identified as Cdtsp2 isolated from the venom of *Crotalus durissus terrificus* (Cdt). The Cdtsp2 fraction originated from the total Cdt venom eluted in the F20 fraction, whose trypsin activity was inhibited (**D**) by N-tosyl-L-phenylalanine-chloromethyl ketone (TPCK) and Nα-tosyl-L-lysinyl-chloromethyl ketone (TLCK). sPLA2 (F16) was inhibited by its natural F3 Crtp inhibitor along with the palmitoyl trifluoromethyl ketone inhibitor (PACOCF3; PCF3 in the figure) and 5-(4-benzyloxyphenyl)-4S-(7-phenylheptanoylamino) pentanoic acid (KH064 in the figure). This effect was not observed for the F20 fraction. Therefore, the biological effects induced by CdtSP2 did not involve contamination with sPLA2 (**C**). However, only one F20 fraction presented a single coagulation display of citrated ox plasma, which was determined following the protocol described by Fonseca et al. [27] (**B**).
